# Acute Beetroot Juice Ingestion Does Not Alter Renal Hemodynamics during Normoxia and Mild Hypercapnia in Healthy Young Adults

**DOI:** 10.3390/nu13061986

**Published:** 2021-06-09

**Authors:** Christopher L. Chapman, Zachary J. Schlader, Emma L. Reed, Morgan L. Worley, Blair D. Johnson

**Affiliations:** 1 Center for Research and Education in Special Environments, Department of Exercise & Nutrition Sciences, University at Buffalo, Buffalo, NY 14214, USA; cchapma3@uoregon.edu (C.L.C.); ereed9@uoregon.edu (E.L.R.); mworley@iu.edu (M.L.W.); 2 Department of Human Physiology, University of Oregon, Eugene, OR 97403, USA; 3 Department of Kinesiology, School of Public Health, Indiana University, Bloomington, IN 47405, USA; zschlade@indiana.edu

**Keywords:** nitrate, nitrite, nitric oxide, beet juice, renal blood flow, renal physiology, carbon dioxide, kidney

## Abstract

Arterial hypercapnia reduces renal perfusion. Beetroot juice (BRJ) increases nitric oxide bioavailability and may improve renal blood flow. We tested the hypothesis that acute consumption of BRJ attenuates both decreases in blood velocity and increases in vascular resistance in the renal and segmental arteries during acute hypercapnia. In fourteen healthy young adults, blood velocity and vascular resistance were measured with Doppler ultrasound in the renal and segmental arteries during five minutes of breathing a carbon dioxide gas mixture (CO_2_) before and three hours after consuming 500 mL of BRJ. There was no difference between pre- and post-BRJ consumption in the increase in the partial pressure of end-tidal CO_2_ during CO_2_ breathing (pre: +4 ± 1 mmHg; post: +4 ± 2 mmHg, *p* = 0.4281). Segmental artery blood velocity decreased during CO_2_ breathing in both pre- (by −1.8 ± 1.9 cm/s, *p* = 0.0193) and post-BRJ (by −2.1 ± 1.9 cm/s, *p* = 0.0079), but there were no differences between pre- and post-consumption (*p* = 0.7633). Segmental artery vascular resistance increased from room air baseline during CO_2_ at pre-BRJ consumption (by 0.4 ± 0.4 mmHg/cm/s, *p* = 0.0153) but not post-BRJ (*p* = 0.1336), with no differences between pre- and post-consumption (*p* = 0.7407). These findings indicate that BRJ consumption does not attenuate reductions in renal perfusion during acute mild hypercapnia in healthy young adults.

## 1. Introduction

The physiological and pathophysiological responses of the kidneys and lungs to maintain essential body homeostasis in health and disease, including acid–base regulation and the handling of carbon dioxide (CO_2_) and bicarbonate, are remarkably integrative [1]. Therefore, it is not surprising that clinical conditions that are associated with chronic or acute elevations in arterial CO_2_ content (i.e., hypercapnia) such as chronic obstructive pulmonary disease (COPD) and sleep apnea are at greater risks of kidney disease and acute kidney injury [1,2,3,4,5,6]. Hypercapnia during the daytime is present in ~14% of patients with sleep apnea [7] and the induction or worsening of hypercapnia is the main risk associated with supplemental oxygen use in patients with an acute exacerbation of COPD [8,9]. Despite the potential pathophysiological effects of hypercapnia on the kidneys in these patients, the physiological response of the renal vasculature to an acute hypercapnic state, independent of changes in oxygen tension, had not been studied until our recent investigation. We reported that in healthy young adults, an acute exposure to mild hypercapnia reduced renal perfusion (interpreted from reductions in renal and segmental artery blood velocity) and increased vascular resistance in the kidneys [10]. As an extension of this study, we sought to examine the potential efficacy of consuming beetroot juice on ameliorating the renal vasoconstrictor responses to acute, mild hypercapnia.

Beetroot juice is an excellent source of antioxidants and micronutrients, including potassium, vitamin C, magnesium, and inorganic nitrate (NO_3_^−^) [11]. Most notably, it is the NO_3_^-^ content of beetroot juice that has received much attention in both the literature and popular culture. NO_3_^−^ ingestion is associated with numerous health benefits, including improvements in exercise performance in healthy populations [11] and patients with COPD [12], although this is not a consistent finding in the latter population [13]. In patients with chronic kidney disease, dietary NO_3_^−^ ingestion has been shown to lower blood pressure and elicit reductions in renal resistive index that are interpreted as a diminished renal vasoconstriction [14]. When dietary NO_3_^−^ is ingested, the enterosalivary pathway within the oral cavity reduces NO_3_^−^ to its bioactive form, nitrite (NO_2_^−^), which then enters the systemic blood circulation [15]. There are a number of pathways in the human body by which NO_2_^−^ can be further reduced to nitric oxide (NO) [15], which exerts vasodilatory effects by diffusing into vascular smooth muscle cells and causing relaxation through activation of soluble guanylyl cyclase and subsequently forming cyclic guanosine monophosphate [16]. As a vasodilator, NO exerts a significant role in renal hemodynamics homeostasis in both normotensive and hypertensive conditions [17]. Furthermore, there is some evidence to support that increased NO attenuates renal sympathetic nerve activity [18], which causes an increased renal blood flow [16]. To our knowledge, the effects of beetroot juice consumption on the response of the renal vasculature to hypercapnia have not been reported. Therefore, as an extension of our previous study [10], we hypothesized that acute consumption of beetroot juice would attenuate both decreases in blood velocity and increases in vascular resistance in the renal and segmental arteries during an acute exposure to mild hypercapnia.

## 2. Materials and Methods

### 2.1. Participants

Fifteen healthy young adults participated in this study. Technical difficulties prohibited data collection in one participant during the post-beetroot juice measurement period. Thus, data are presented from 14 participants with the following characteristics: 7 females, 7 males; age: 25.6 ± 3.2 years; height: 169 ± 9 cm; weight: 65.1 ± 11.0 kg; and body mass index: 22.7 ± 3.0 kg/m^2^ (4 out of fourteen participants had a body mass index between 25.0 and 27.9 kg/m^2^). Thirteen of these participants were included in our previous study [10]. Participants were free of any pre-existing autonomic, cardiovascular, metabolic, respiratory, endocrine, and/or kidney disease. Additionally, participants were not on medications and were not smokers. Female subjects were tested during the first 10 days of their self-identified menstrual cycle and were confirmed to be not pregnant via a urine pregnancy test.

### 2.2. Instrumentation and Measurements

Height and nude body weight were measured with a stadiometer and scale (Sartorius, Bohemia, NY, USA). Urine-specific gravity was measured in duplicate with a refractometer (Atago USA, Bellevue, WA, USA). A capnograph (Nonin Medical, Inc., Plymouth, MN, USA) was used to measure the partial pressure of end-tidal CO_2_ (PETCO_2_) and index changes in arterial CO_2_ content. Heart rate was continuously measured via three-lead ECG (DA100C, Biopac Systems, Goleta, CA, USA). The Penaz method was used to measure beat-to-beat blood pressure via finger photoplethysmography (Finometer Pro, FMS, Amsterdam, The Netherlands) from the middle finger of the left hand. Beat-to-beat blood pressure was calibrated to brachial artery blood pressure in the supine position using return-to-flow [19] and was confirmed intermittently by auscultation of the brachial artery with electrosphygmomanometry (Tango M2; SunTech Medical, Raleigh, NC, USA). Blood pressure data from the beat-to-beat finger photoplethysmography technique are reported.

Doppler ultrasound-derived measures of renal and segmental artery hemodynamics were obtained in the supine position using the coronal approach (GE Vivid-Q, Chicago, IL, USA). We have previously described this technique in detail, where blood velocity of the renal vasculature was measured in the distal segment of the right renal artery (renal artery) and the mid-point of a segmental artery (segmental artery) [10,20,21,22,23,24]. The same segmental artery, for a given subject, was used throughout the experimental protocol. Blood velocity was measured across three consecutive cardiac cycles during which participants were instructed to perform a mid-exhalation, non-Valsalva breath hold lasting no more than 10 s [22]. To minimize transient increases in PETCO_2_ associated with breath holding, blood velocity was measured in the segmental artery only during each minute of CO_2_ breathing, whereas renal artery blood velocity was not measured during each minute of CO_2_ breathing. Both renal and segmental artery blood velocity were measured during room air breathing and at the end of five minutes of breathing CO_2_. The same sonographer obtained all renal measurements (C.L.C.). Additionally, the location for the ultrasound transducer was marked on the participant with indelible ink. The transducer was held in place (i.e., was not removed) during all measurements before beetroot juice consumption and after consumption. However, the transducer was removed during the three-hour period between pre- and post-beetroot juice measurements. This approached yielded a within-subject test–retest coefficient of variation of 4.1 ± 1.8% (renal artery) and 5.7 ± 1.8% (segmental artery). The strengths and limitations of this approach have been documented in detail elsewhere [22]. In brief, changes in blood velocity in the renal vasculature were interpreted to reflect changes in blood flow in these conduit vessels [22,25,26,27].

### 2.3. Experimental Protocol

Participants were instructed to abstain from antibacterial mouthwash and chewing gum the morning of this study to avoid interference with the reduction NO_3_^−^ to NO_2_^−^ [28,29,30,31]. Additionally, participants reported to the laboratory after abstaining from caffeine, alcohol, and exercise for 12 h and food for two hours. The start time of each experimental trial was within the same two-hour window for all participants (09:30–11:30 a.m.). Upon arrival, euhydration was confirmed via assessment of a spot urine sample with a specific gravity <1.020. Then, participants assumed a supine position and were instrumented accordingly. Following 45 min of supine rest, pre-beetroot juice baseline hemodynamic and renal measurements were obtained while participants breathed room air through a mouthpiece connected to a four-way valve (Air). Then, participants were switched to breathing a CO_2_ gas mixture (CO_2_) consisting of 3% CO_2_, 21% O_2_, and 76% N_2_. Participants breathed CO_2_ for five minutes with renal measurements and hemodynamics obtained every minute (as described above). Following completion of all pre-beetroot juice consumption measurements, participants consumed 500 mL of a commercially available beetroot juice (Biotta^®^ Beet Juice, Fishers, IN, USA) within 5 min. The 500 mL is the drink volume that is commercially available, which we used to improve our external validity, and resulted in an overall mean dose for participants of 7.9 ± 1.3 mL of beetroot juice per kilogram of body mass. This beetroot juice and dose has been used in previous studies [30,31] and has an on-label nutrient profile of 0 g fat, 24 g carbohydrate, 3 g protein, and 95 mg sodium. Following beetroot juice consumption, participants rested quietly in the laboratory for two hours and 15 min. During the break, participants were given 250 mL water to consume to reduce thirst perception and eliminate any perceived aftertaste of the beetroot juice. Participants were instructed to finish consuming this water at least 30 min prior to beginning supine rest and were not allowed any other additional food or beverage items. Participants were also allowed to void their bladder during this break period to reduce discomfort during post-consumption measurements. After the two hours and 15 min break, participants assumed the supine position and rested quietly for 45 min. After supine rest, post-beetroot juice measurements during room air and CO_2_ breathing occurred following the same procedures as pre-beetroot juice consumption. This timing was specifically designed so that post-beetroot juice measurements occurred exactly three hours after beetroot juice consumption, which has been documented by others as the time by which blood NO_2_^−^ concentrations peak [30,32,33].

### 2.4. Data and Statistical Analyses

A data acquisition system (Biopac MP150; Goleta, CA, USA) was used to continuously sample PETCO_2_ (15.6 Hz), heart rate (1000 Hz), and mean arterial pressure (62.5 Hz). To minimize the influence of the breath hold procedure on PETCO_2_ measurements, PETCO_2_ data were extracted as the average during the 45 s period immediately prior to the renal measurements (i.e., PETCO_2_ was extracted during normal breathing only). Heart rate and mean arterial pressure data were extracted during the same cardiac cycles as the renal measurements, as described above. Stroke volume was estimated by Modelflow [34]. Cardiac output was calculated as the product of heart rate and stroke volume. The quotient of mean arterial pressure and cardiac output was used to calculate total peripheral resistance. Vascular resistance in the renal and segmental arteries were calculated as the quotient of mean arterial pressure and blood velocity.

Segmental artery blood velocity, vascular resistance, and all hemodynamic variables are reported as *n* = 14 (7 females and 7 males). Due to acoustic shadowing of the kidney occurring during CO_2_ breathing pre-beetroot juice consumption (*n* = 1) or post-beetroot juice consumption measurements (*n* = 3), renal artery blood velocity and vascular resistance are reported as *n* = 10 (4 females and 6 males) for renal artery comparisons involving CO_2_ and *n* = 11 (5 females and 6 males) for renal artery comparisons involving the effects of beetroot juice independent of CO_2_. Data were analyzed with Prism software (version 9.1, GraphPad Software, La Jolla, CA, USA). Normality was confirmed using the Shapiro–Wilk test. Hemodynamic and renal vascular responses to beetroot juice consumption, independent of CO_2_ breathing, were analyzed with a two-tailed paired *t*-test. Two-way repeated-measures ANOVAs were used to compare the effect of beetroot juice (condition) on hemodynamic and renal vascular responses during 5 min of CO_2_ breathing (time). When an ANOVA revealed a significant *F* statistic, post hoc Dunnett’s tests were used to compare changes during CO_2_ from Air baseline (i.e., 0 min) and post hoc Sidak’s tests were used to compare differences between pre-and post-beetroot juice consumption during CO_2_ breathing. Statistical significance was set a priori at *p* ≤ 0.05. Actual *p*-values are reported where possible. Data are reported as the means ± SD.

## 3. Results

### 3.1. Effect of Beetroot Juice Consumption during Room Air Breathing

**Figure 1:**
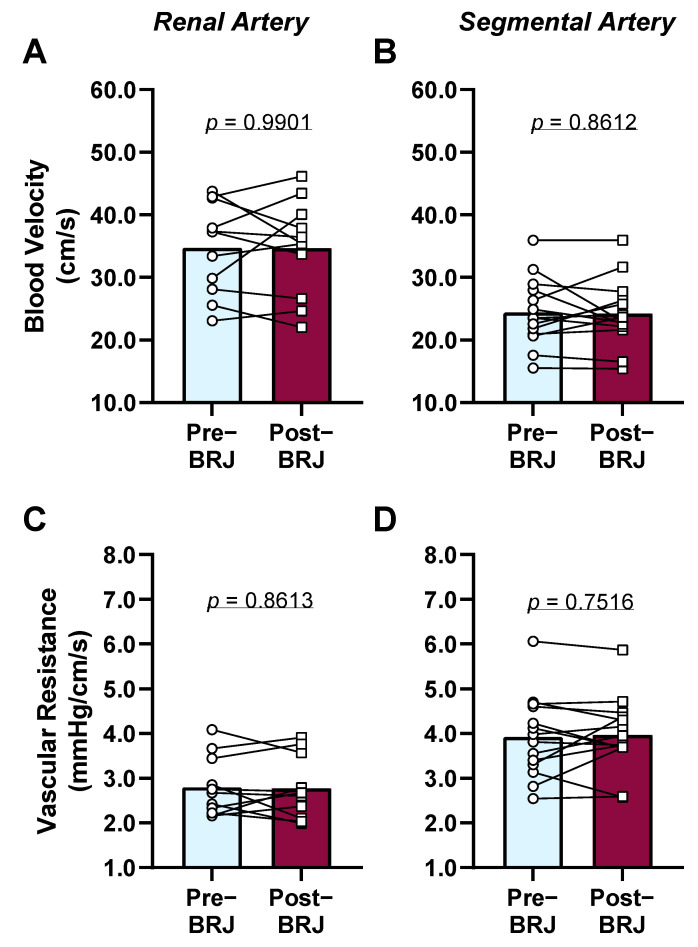
Effect of beetroot juice (BRJ) at three hours post-consumption on renal (panels **A** and **C**, *n* = 11, 5 females and 6 males) and segmental (panels **B** and **D**, *n* = 14, 7 females and 7 males) artery blood velocity and vascular resistance during room air breathing. Data were analyzed using a two-tailed paired *t*-test and are presented as the means (bar) with individual values.

There was no effect of beetroot juice on any hemodynamic variables (*p* ≥ 0.1021, Table 1). Additionally, beetroot juice consumption did not change blood velocity or vascular resistance in the renal and segmental arteries (*p* ≥ 0.7516, Figure 1).

### 3.2. Effect of Beetroot Juice Consumption during CO_2_ Breathing

#### 3.2.1. PETCO_2_ and Cardiovascular Responses

**Figure 2:**
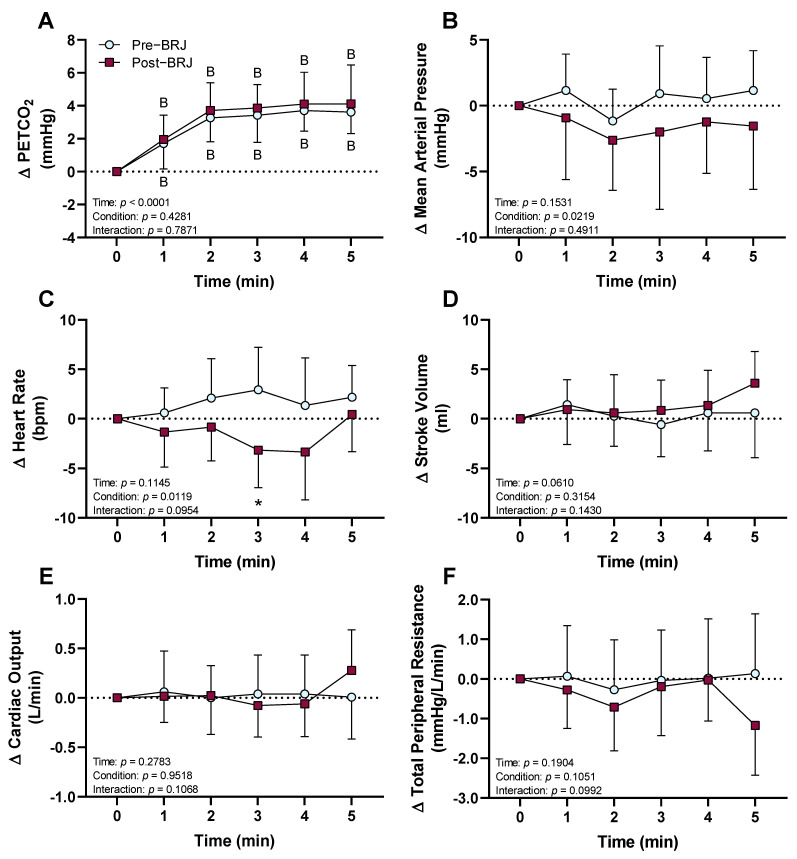
Effect of beetroot juice (BRJ, condition) at three hours post-consumption on the hemodynamic response to five minutes of CO_2_ breathing (time). Data are presented as the means ± SD as the change from room air baseline (0 min). Data were analyzed using a two-way repeated-measures ANOVA with post hoc Dunnett’s tests to compare changes during CO_2_ from room air baseline and post hoc Sidak’s tests to compare differences between pre-and post-beetroot juice consumption during CO_2_ breathing. (**A**) PETCO_2_: partial pressure of end-tidal CO_2_; (**B**) mean arterial pressure; (**C**) heart rate; (**D**) stroke volume; (**E**) cardiac output (**F**) total peripheral resistance. *n* = 14 (7 females and 7 males). ^B^ different from room air baseline (*p* ≤ 0.0074); * different from pre-beetroot juice consumption (*p* = 0.0539).

PETCO_2_ increased from baseline during CO_2_ breathing in both pre- and post-beetroot juice, and there were no differences between pre- and post-beetroot juice consumption (*p* = 0.4281, Figure 2A). There was a significant main effect of beetroot juice for mean arterial pressure (*p* = 0.0219), indicating a decrease in mean arterial pressure throughout CO_2_ breathing post-beetroot juice consumption compared to pre-beetroot juice consumption. However, post hoc multiple comparisons analysis did not reveal differences between pre- and post-beetroot juice consumption at any timepoint during CO_2_ breathing (*p* ≥ 0.1342, Figure 2B). Heart rate was decreased at three minutes of CO_2_ breathing with post-beetroot juice consumption compared to pre-beetroot juice consumption (*p* = 0.0539), but there were no differences between trials at any other timepoint (*p* ≥ 0.1268, Figure 2C). There were no differences between pre- and post-beetroot juice consumption in stroke volume (*p* = 0.3154, Figure 2D), cardiac output (*p* = 0.9518, Figure 2E), and total peripheral resistance (*p* = 0.1051, Figure 2F).

#### 3.2.2. Segmental Artery Hemodynamics

**Figure 3:**
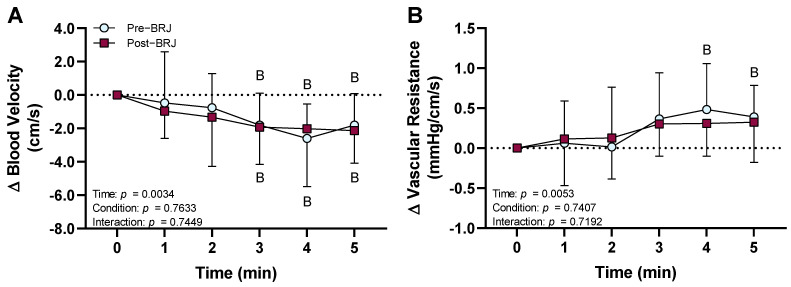
Effect of beetroot juice (BRJ, condition) at three hours post-consumption on blood velocity and vascular resistance in the segmental artery during CO_2_ breathing (time). Data are presented as the means ± SD as the change from room air baseline (0 min). Data were analyzed using a two-way repeated-measures ANOVA with post hoc Dunnett’s tests to compare changes during CO_2_ from room air baseline and post hoc Sidak’s tests to compare differences between pre- and post-beetroot juice consumption during CO_2_ breathing. (**A**) segmental artery blood velocity; (**B**) segmental artery vascular resistance. *n* = 14 (7 females and 7 males). ^B^ different from room air baseline (*p* ≤ 0.0394).

Segmental artery blood velocity decreased from room air baseline during 3–5 min of CO_2_ breathing at both pre- and post-beetroot juice consumption (*p* ≤ 0.0348), but there were no differences in the magnitude of these decreases between pre- and post-beetroot juice consumption (*p* = 0.7633, Figure 3A). Segmental artery vascular resistance increased from room air baseline during 4–5 min of CO_2_ breathing at pre-beetroot juice consumption (*p* ≤ 0.0394, Figure 3B). However, there were no changes from room air baseline during CO_2_ breathing at post-beetroot juice consumption (*p* ≥ 0.0695), and there were no differences between pre- and post-beetroot juice consumption throughout CO_2_ (*p* = 0.7407, Figure 3B).

#### 3.2.3. Renal Artery Hemodynamics

**Figure 4:**
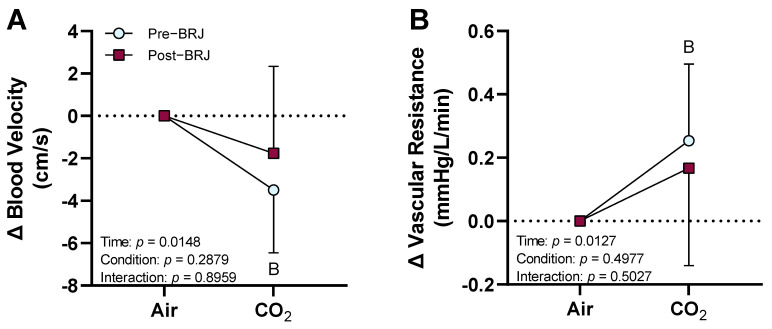
Effect of beetroot juice (BRJ, condition) at three hours post-consumption on blood velocity and vascular resistance in the renal artery during five minutes of CO_2_ breathing (time). Data are presented as the means ± SD as the change from room air baseline. Data were analyzed using a two-way repeated-measures ANOVA with post hoc Sidak’s tests to compare changes from room air baseline and to compare differences between pre-and post-beetroot juice consumption during CO_2_ breathing. (**A**) renal artery blood velocity; (**B**) renal artery vascular resistance. *n* = 10 (4 females and 6 males). ^B^ different from room air baseline (*p* ≤ 0.0177).

Renal artery blood velocity decreased from room air baseline during CO_2_ breathing at pre-beetroot juice consumption (*p* = 0.0093) but not post-beetroot juice consumption (*p* = 0.4113, Figure 4A). There were no differences in the magnitude of change in blood velocity between pre- and post-beetroot juice consumption (*p* = 0.2879, Figure 4A). Renal artery vascular resistance increased from room air baseline during CO_2_ breathing at pre-beetroot juice consumption (*p* = 0.0177), but not post-beetroot juice consumption (*p* = 0.2645, Figure 4B). There were no differences between pre- and post-beetroot juice consumption following CO_2_ breathing (*p* = 0.4977, Figure 4B).

## 4. Discussion

Contrary to our hypothesis, the findings from the present study do not support that acute beetroot juice consumption attenuates decreases in blood velocity or diminishes increases in vascular resistance in the renal and segmental arteries during an acute exposure to mild hypercapnia in healthy young adults. Rather, we found similar reductions in segmental artery blood velocity during CO_2_ breathing between pre- and post-beetroot juice consumption. Additionally, we did not observe differences between pre- and post-beetroot juice during CO_2_ in segmental artery vascular resistance. We did observe increases from room air baseline in pre- but not post-beetroot juice. In the renal artery, we did not observe differences in blood velocity and vascular resistance between pre- and post-beetroot juice during CO_2_. However, pre-beetroot juice resulted in decreased blood velocity and increased vascular resistance during CO_2_ compared to room air baseline, but there were no differences from room air baseline during CO_2_ at post-beetroot juice.

We hypothesized that acute beetroot juice consumption would attenuate both decreases in blood velocity and increases in vascular resistance in the renal and segmental arteries during an acute, mild hypercapnic state. Although the mechanisms are not fully understood, the kidneys are acutely and dynamically sensitive to mild changes in arterial CO_2_ content likely owing to increases in renal sympathetic nerve activity [10]. It has been previously suggested that increasing NO bioavailability influences renal blood flow [16], based on evidence indicating that NO inhibition, via *N*-methyl-L-arginine infusion, increased renal sympathetic nerve activity in rats [18]. Along these lines, beetroot juice consumption increases NO bioavailability via the NO_3_^−^–NO_2_^−^–NO pathway, which is thought to become more pronounced during regional ischemia to augment the l-arginine–NO synthase pathway [15]. In dogs, a more profound normoxic-hypercapnic stimulus (i.e., arterial CO_2_ partial pressure ~80 mmHg) reduced renal blood flow by ~20% [35] whereas the mild normoxic-hypercapnic stimulus (i.e., PETCO_2_ of 48 ± 3 mmHg) in the present study reduced segmental artery blood velocity by 8%. That beetroot juice consumption did not improve renal perfusion during mild hypercapnia may be a result of an insufficient duration or magnitude (i.e., the severity of the hypercapnia) of stimulus to elicit renal ischemia. Therefore, the NO_3_^−^–NO_2_^−^–NO pathway may not have been promoted to influence renal perfusion. This speculation may explain why statistically significant increases in segmental artery vascular resistance were observed during CO_2_ breathing at pre-beetroot juice consumption, but not post-beetroot juice consumption. Additionally, whether these acute effects reflect the response of the renal vasculature to hypercapnia with chronic beetroot juice consumption remains unknown. Moreover, beetroot juice may be less efficacious in young, healthy adults during acute perturbations in arterial CO_2_ content compared to a clinical population. Therefore, future studies examining the potential efficacy of beetroot juice in adults at risk for exacerbations of hypercapnia (i.e., COPD and sleep apnea) remain warranted. There are concerns for kidney stone formation in patients with chronic kidney disease due to the high oxalic acid composition of beetroot juice [36]. However, there may be potential benefits in patients with chronic kidney disease, as acute dietary beetroot juice supplementation improved exercise capacity [37] and chronic supplementation lowered blood pressure and reduced renal resistive index [14]. While the beetroot juice did not alter renal perfusion in the present study, there are likely other bioactive compounds that may be beneficial for overall health, including betaine, betacyanins, betanins, polyphenols, flavonoids, vitamins and minerals [36].

An interesting finding in the present study was that there was a significant main effect of beetroot juice consumption for a reduced mean arterial pressure during CO_2_ breathing (Figure 2B) despite no changes in mean arterial pressure during room air breathing (i.e., normoxic conditions). Thus, these data indicate that there may be other beneficial responses of beetroot juice consumption for patients at risk of hypercapnia due to COPD or sleep apnea. Given the links between hypertension and both sleep apnea [38] and COPD [39], the data from the present study support further research in this area regarding the efficacy of beetroot juice supplementation to improve blood pressure in these patient populations.

The present study has several limitations that are worth discussing. First, we did not include a time control group. Thus, we are unable to confirm that our findings are not a result of the three hours that elapsed between testing periods. However, despite this important limitation, efforts were made to improve scientific rigor, including having the participants rest quietly during this break period. Second, the Doppler ultrasound measures of renal and segmental artery blood velocity were interpreted to reflect changes in renal blood flow in conduit vessels in the kidney. The findings of the present study may potentially differ when using techniques to quantify renal plasma flow, such as para-aminohippurate clearance, which involves quantifying the complete circulation throughout the kidneys [40]. That said, the Doppler ultrasound technique employed in the present study was determined to be advantageous compared to clearance techniques because of the ability of Doppler ultrasound to capture rapid, dynamic changes in renal hemodynamics [22]. Third, we did not measure NO_2_^−^ in the saliva or blood. Participants were specifically instructed to abstain from antibacterial mouthwash and chewing gum the morning of this study, as they have successfully increased circulating NO_2_^−^ according to previous reports [29,30,31]. These products have been previously shown to profoundly attenuate the ability of the enterosalivary pathway to reduce NO_3_^−^ into NO_2_^−^ in the oral cavity, and therefore, attenuate increases in blood NO_2_^−^ concentration [28]. Previous reports using the same commercially available beetroot juice product and the same 500 mL dose as the present study have reported that blood NO_2_^−^ increased by ~200 nM at three hours post-consumption in healthy young adults [30]. Additionally, some studies have given beetroot juice to participants following an overnight fast [30,33]. Future studies are warranted to investigate whether the renal hemodynamic responses to beetroot juice consumption differs following a prolonged fast. Fourth, we did not determine if there is a dose–response effect of acute beetroot juice ingestion on renal hemodynamics.

In conclusion, our findings revealed that in healthy young adults, acute beetroot juice consumption does not attenuate decreases in blood velocity or increases in vascular resistance in the renal and segmental arteries during an acute exposure to mild hypercapnia. Our data also indicate that acute beetroot juice consumption does not increase blood velocity or decrease vascular resistance in the renal and segmental arteries during resting normoxic conditions.

## Figures and Tables

**Table 1:** Effect of beetroot juice consumption during room air breathing.

Parameter	Pre-Beetroot Juice	Post-Beetroot Juice	*p*-Value
PETCO_2_ (mmHg)	45 (3)	45 (3)	0.8557
Mean arterial pressure (mmHg)	91 (5)	92 (7)	0.3929
Heart rate (bpm)	61 (6)	63 (9)	0.2413
Stroke volume (mL)	93 (14)	88 (18)	0.1689
Cardiac output (L/min)	5.7 (0.8)	5.4 (0.9)	0.2234
Total peripheral resistance (mmHg/L/min)	16.4 (2.6)	17.7 (3.1)	0.1021

PETCO_2_: partial pressure of end-tidal CO_2_. Data were analyzed using a paired *t*-test and are presented as the mean (SD). *n* = 14 (7 females and 7 males).

## Data Availability

The data from the current study are not publicly available, but are available from the corresponding author upon reasonable request.
